# Evaluation the association of facet tropism in multi-sports athletes with cervical disc hernia

**DOI:** 10.1186/s12891-022-05552-x

**Published:** 2022-06-27

**Authors:** Ali Eroglu, Ahmet Eroglu

**Affiliations:** 1 Department of Sports Medicine, Erenköy Physical Medicine and Rehabilitation Hospital, Şemsettin Günaltay Cad. Sultan Sok No:14, 34736 Kadıköy/Istanbul, Turkey; 2 Department of Neurosurgery, Haydarpaşa Sultan Abdülhamid Education and Research Hospital, Istanbul, Turkey

**Keywords:** Cervical facet angle, Multi-sports athletes, Cervical disc hernia

## Abstract

**Background:**

Facet tropism (FT) can be defined as the angular difference between the orientation of the right and left facet joints in axial or sagittal planes. Most studies discuss about the relationship with lumbar disc hernia and facet joint angle. However, little is known about the association of facet tropism with disc herniation in the cervical spine in multisports athletes. In this study, We aimed to investigate the relationship between cervical facet tropism and disc hernia in athletes of different branches between the ages of 20–40 from the cervical MR images of the cases.

**Methods:**

This is a retrospective study performed on athletes who applied our hospital between January 2014–2019 with neck pain and have MR imaging of the cervical spine. Cervical MR images of the patients were evaluated by an experienced radiologist from the hospital system database and archives. 79 cases (52 men and 27 women) were included in the study.

**Results:**

No statistically significant difference was found between the facet joint angles of both groups at all levels (*p*˃0.05). Only left C6-7 disc angles of CDH group were measured as 92.99° ± 10.77^0^ (62^0^–113^0^) and 88.58° ± 7.65° (67°-110°) for the normal group and this difference was found statistically significant (*p* = 0.007).

**Conclusion:**

In this study, we did not predict that cervical facet tropism may be a factor associated with cervical disc hernia in young athletes with CDH.

## Background

Facet joints play an important role in the posterior stabilization of the vertebrae through the upper and lower joint faces. Facet joints carry about a third of the dynamic load on the vertebrae, preventing excessive rotation, flexion and translation [1]. Facet tropism (FT) can be defined as the angular difference between the orientation of the right and left facet joints in axial or sagittal planes. Until now, mostly the relationship between the lomber FT and lumbar disc hernia, spondylolisthesis and degenerative disc disease has been investigated in different populations [2–5].

However, there were limited articles investigating cervical facet joint tropism and the relationship between cervical disc hernia (CDH) [6, 7]. And also; There was no data regarding the relationship between the facet tropism and the CDH for the multi-sport athletes. Therefore, we aimed to investigate the relationship between cervical facet tropism and disc hernia in athletes of different branches between the ages of 20–40 from the cervical MR images of the cases.

## Materıal and method

This is a retrospective study performed on athletes who applied our hospital; between January 2014–2019 with neck pain and have MR imaging of the cervical spine. A total of 79 cases, 41 of whom were cervical disc hernias were included in our study. 33 of our cases were rowing, 17 were archery, 14 were fencing, 15 were gymnasts. CDH cases evaluated as bulging and protruding as a result of MR evaluations were included in the study. All cases aged 20–40 years with a diagnosis of CDH and bilateral facet joint angles at C4-5, C5-6 and C6-7 levels measured on cervical magnetic resonance imaging (MRI) tests. In the comparison of the two facet joint angles, a difference of < 7° was classified as no tropism, 7°-14° as moderate tropism and > 14° as severe tropism. Before the data collection, Instıtuonal review board (IRB) had taken by our hospital's ethics Committee (07/01/2014–41). Cervical MR images of the patients were evaluated by an experienced radiologist from the hospital system database and archives. 79 cases (52 men and 27 women) were included in the study. The cases were divided into two groups as CDH and Normal group. While the mean age of CDH cases was 32.49 ± 4.79 (23–40), the average age of the normal group cases was 32.42 ± 4.46 (22–40). Those with a history of spinal surgery, trauma, chronic inflammatory disease, recovery or presence of spinal malignancy and congenital vertebral anomalies were excluded. 41 (29 m, 12 f) patients with a single or double level, and left or right side herniated posterolateral CDH at the C4-C5, C5-C6 or C6-C7 discs were included in this study as a CDH group. In the study, 38 participants (22 m and 16 f) who came to the clinic for routine examination were included as a control group. The control group also consisted of randomly selected healthy individuals who applied to the clinic for routine examination from the hospital database and who had a cervical MRI as a result of the physical examination.

### MR images

MRI of the cervical spine was performed using a 3-dimensional (3D) spoiled gradient-echo sequence with 6 asymmetric echoes was used on a 1.5-T MRI system (Signa HDxt; GE Healthcare, Milwaukee, Wis). And also the MR unit used an 8-channel phase array spine coil with the following imaging parameters: repetition time (TR), 12.8 ms; echo time (ET), 1.6 to 10.1 ms; slice thickness, 4 mm; 16 to 20 slices in 3D slab; flip angle, 5 degrees; bandwidth, 125 kHz; field of view (FOV), 44 cm; matrix, 224 × 160; and total scan time, 12 s.

### Cercival facet angle measurement

**Fig. 1:**
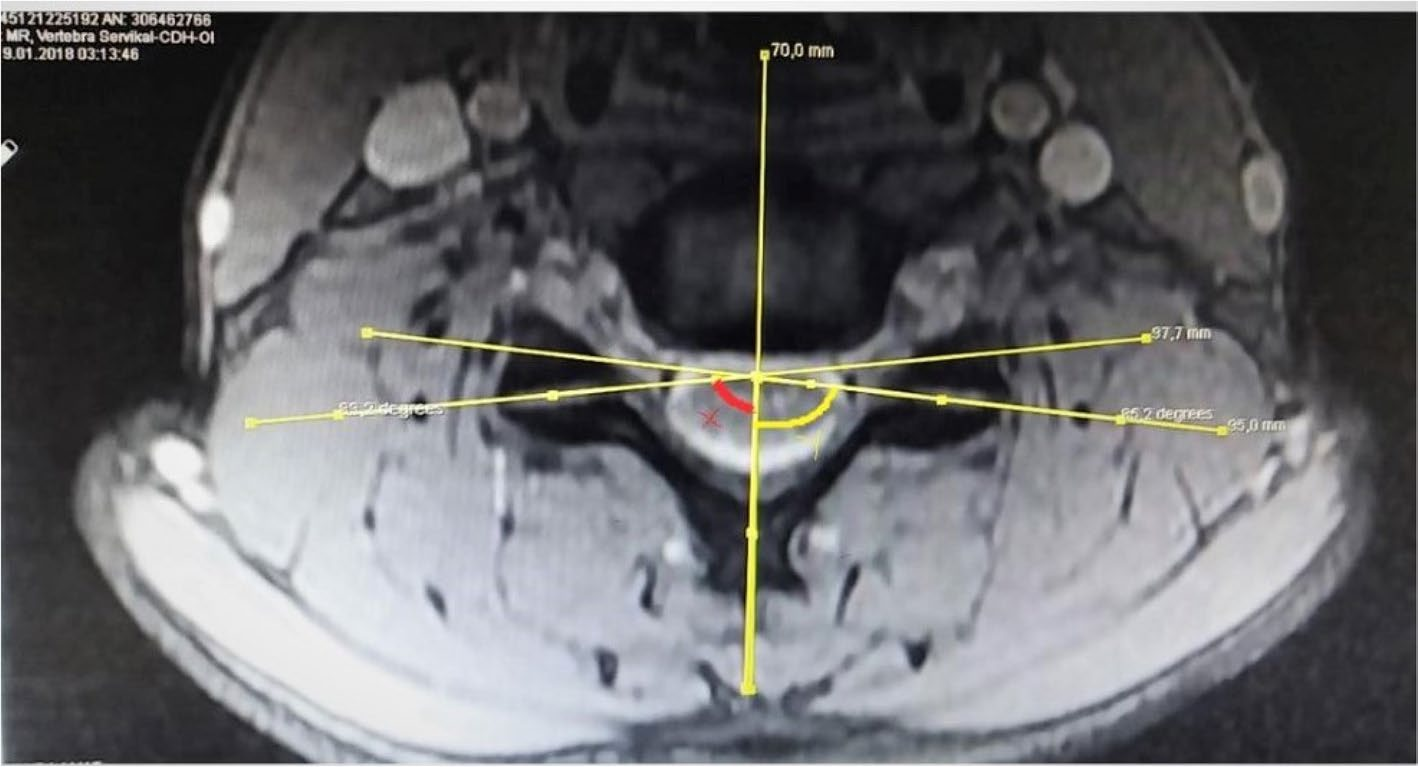
Reference line (RL) was accepted as the line passing through the center of the disc and the middle of the processus spinosus. The facet line (FL) was defined as the line drawn between the anteromedial and posteromedial edges of the upper joint facets on both sides. The angle between the two lines was measured and defined as the facet joint angle on both sides

Measurements of axial facet joint angles at the level of C4-5, C5-6 and C6-7 were made according to the method previously described by Xu et al. [8]. Reference line (RL) was accepted as the line passing through the center of the disc and the middle of the processus spinosus. The facet line (FL) was defined as the line drawn between the anteromedial and posteromedial edges of the upper joint facets on both sides. The angle between the two lines was measured and defined as the facet joint angle on both sides (Fig. 1). A radiologist and a neurosurgeon measured the angles with an automatic goniometer in the system. The arithmetic average of two separate measurements was taken and the main angle was obtained.

### Statistical analyses

As a statistical method in the analysis of data in the research; descriptive analysis (mean, standard deviation, minimum, maximum) were used. In the analysis of continuous data, compatibility to normal distribution was found using the Kolmogorov–Smirnov test. Chi square test, t test and covariance analysis were applied due to the compliance with normal distribution. The results were evaluated within the 95% confidence interval and *p* < 0.05 was considered significant.

## Results

**Fig. 2:**
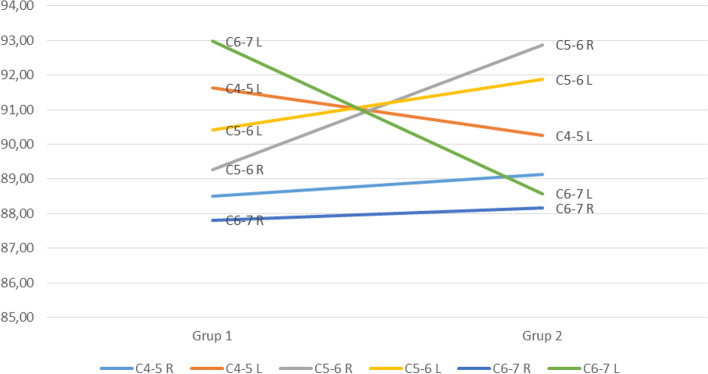
Representation of facet angle values of both groups

A total of 79 cases, 41 of whom were cervical disc hernias were included in our study. 33 of our cases were rowing, 17 were archery, 14 were fencing, 15 were gymnasts. 8 of the cases with cervical disc hernias were on the C4-5 disc, 22 were on the C5-C6 disc and 11 were on the C6-C7 disc level. CDH cases evaluated as bulging and protruding as a result of MRI evaluations were included in the study. Almost all the disc hernias of the patients were posterolateral as an anatomical location, 24 were on the right side and 17 were on the left side. Demographic features and facet joint angles of the CDH and normal groups are shown in Table 1 and Fig. 2. Table 2 shows the distribution of cases according to the gender. In terms of age, the distribution of the CDH group was 32.49 ± 4.79 (23–40) and the distribution of the normal group was 32.42 ± 4.46 (22–40). The difference between the two groups was not statistically significant (*p* = 0.933). In terms of height, the distribution of the CDH group was 176.95 ± 5.66 cm (165–188) and the normal group distribution was 174.89 ± 6.54 cm (165–189), but the difference between the two groups was not statistically significant (*p* = 0.095). While the distribution of CDH cases in terms of weight was 77.12 ± 8.86 kg (56–90), the weight distribution of the normal group was found to be 72.94 ± 9.97 kg (57–90). The difference between the two groups was statistically significant (*p* = 0.027). The average weight of the normal group was low.

**Table 1:** Physical characteristics and bilateral facet angle degrees of CDH and normal groups

	CDH	Normal	p
AGE	32,49 ± 4,79 (23–40)	32,42 ± 4,46 (22–40)	0,93
ANGLE	176,95 ± 5,66 (165–188)	174,89 ± 6,54 (165–189)	0,09
WEIGHT	77,12 ± 8,86 (56–90)	72,94 ± 9,97 (57–90)	0,03
BMI	24,61 ± 3,17	23,82 ± 2,93	0,53
C4_5R	88,5 ± 8,78 (73–113)	89,13 ± 6,79 (74–101)	0,59
C4_5L	91,63 ± 8,39 (77–115)	90,26 ± 7,28 (70–108)	0,66
C5_6R	89,28 ± 8,56 (68–107)	92,88 ± 7,68 (77–118)	0,09
C5_6L	90,41 ± 10,94 (61–117)	91,87 ± 8,62 (79–125)	0,50
C6_7R	87,81 ± 6,71 (69–100)	88,16 ± 8,06 (69–105)	0,90
C6_7L	92,99 ± 10,77 (62–113)	88,58 ± 7,65 (67–110)	0,01

**Table 2:** Gender distribution of cases

		CDH group		Normal group	
		n	n%	n	n %
GENDER	M	29	56,9%	22	43,1%
	F	12	42,9%	16	57,1%

No statistically significant difference was found between the facet joint angles of both groups at all levels (p˃0.05). Only left C6-7 facet joint angles of CDH group were measured as 92.99° ± 10.77° (62°-113°) and 88.58° ± 7.65° (67°-110°) for the normal group and this difference was found statistically significant (*p* = 0.007). After the weight variable was taken under control, the left C6-7 value of the CDH group was found to be 92,673 kg and the normal group value was 88,916 kg (CDH group values without the weight control were 92.99 kg normal group value 88.58 kg) and the difference between the two groups was not statistically significant (*p* = 0.17). Without taking into account the weight (covariant), we see that the left C6-7 value has a significant effect between the two groups. But when the weight (covariant) is taken into account, we see that the left C6-7 value has no significant effect between the two groups. In other words, left C6-7 values of the two groups are similar (Table 3).

**Table 3:** Facet angle values between two groups when weight (covariant) is taken into account

Weight = 75,1127	Mean	Std. Error	95% Confidence Interval	
			Lower Bound	Upper Bound
CDH	92,673	1,477	89,731	95,615
Normal	88,916	1,536	85,857	91,975

## Dıscussıon

The hyaline cartilage and facet joints above the subchondral bone are the only synovial joints in the spine that help carry 16% of the vertical load on the intervertebral discs [9].

Facet joints provide rear mechanical support to protect the discs from excessive rotations in flexion and extension by fixing the movement segment. We evaluated that athletes in archery, fencing, rowing and gymnastics branches where cervical disc and facet joints are used more often, such as hyperflexion, hyperextension, lateral bending and cervical rotation, would be more suitable for our study.

Although facet joint tropism means asymmetry of the left and right facet joints, the criteria for defining it are very different. While Noren et al. defined facet asymmetry as an angle difference of > 5° bilaterally [10], Huang et al. accepted the tropism at cervical facet level > 7° [11]. In other biomechanical studies, facet asymmetry was evaluated that facet joint angles greater than 1° –10°[12, 13]. According to the study results available in the literature, there is no definitive consensus about the angle of servical facet joint tropism.

Unlike the lumbar spine, the facet direction of the cervical spine is more complex. While the facet joints are located more vertically in the lumbar spine, they are located more axially and coronal in the cervical region [14]. In previous studies, it has been mentioned that facet tropism can cause unbalanced stress distribution in the disc, thus causing disc degeneration, and even disc herniation [4, 15, 16]. Literature studies mostly focus on the relationship between facet joint tropism, low back or neck problems, degeneration of the disc, facet joint, and spondylolysis [7, 8, 17–20]. In most of these studies, cases are older age, patients who do not exercise regularly and have a low back pain. As far as we know, our study will be the first with the control group evaluating the association of cervical facet joint tropism and cervical disc hernia in multisport athletes aged 20–40 in 4 different sports activity. In a study by Xin Rong et al.; in patients over 50 years of age, it was evaluated that there was a relationship between disc degeneration and tropism, and it was shown that facet tropism was mostly at C2-3 level [16]. In the same study, it has been experimentally demonstrated that the facet joint and intervertebral disc with facet tropism are exposed to higher pressures than the symmetrical model.

Therefore, we aimed to investigate whether abnormal stress on joints and discs with facet tropism would be related to disc herniation. However; In a study conducted by Okada et al. on 223 asymptomatic healthy cases with a 10-year retrospective MRI study, no correlation other than age was found in the development of disc degeneration [21]. However, anatomical factors related to the patients were not adequately shed lighted on this study. In individuals with asymmetrical facet tropism, has been shown an increase of 49.2% by flexion, 57.1% by extension and 30.6% by axial rotation [16]. Based on this study, it can be suggested that facet joint tropism may be an anatomical risk factor in the development of cervical disc herniation. In our study, when compared the cervical disc hernia group and the normal group, we could not find a statistically significant facet tropism. In the literature, we could not find a study evaluating the correlation between facet tropism and cervical disc hernia in athletes. Another study supporting ours by D. Y. Lee et al. demonstrated that facet tropism has not played a role in the development of lumbar disc herniation in adolescents [22]. On the other hand, a retrospective study by Huang X et al. with 96 cases over the age of 50 years, found a correlation between cervical disc hernia and tropism [6]. The cases in our study were in the younger (20–40) age range and consisted of athletes with regularly trained. We evaluated the MRI findings with bulding and protrusion. In the mentioned study, it was not stated at which level disc cases were included. As cervical disc degeneration shows only age-related development, the association of facet tropism with cervical disc hernia may be in older ages [8].

This study had several limitations. First, we think the number of cases is insufficient. In addition, there is no clear consensus of what is the standard cervical vertebral facet tropism. In the future, more studies with more patients and multicentre studies are needed to define the angle of facet tropism clearly.

## Conclusıon

In this study, we did not predict that cervical facet tropism may be a factor associated with cervical disc hernia in young athletes with CDH. Therefore, evaluation of hernia development in young ages may be a separate study in the cases with facet tropism who makes regular sports and exercise.

## Data Availability

The datasets used and/or analysed during the current study available from the corresponding author on reasonable request.

## References

[1] Yang KH, King AI (1984). Mechanism of facet load transmission as a hypothesis for low-back pain. Spine.

[2] Chadha M, Sharma G, Arora SS (2013). Kochar, V: Association of facet tropism with lumbar disc herniation. Eur Spine J.

[3] Do DH, Taghavi CE, Fong W, Kong MH, Morishita Y, Wang JC (2011). The relationship between degree of facet tropism and amount of dynamic disc bulge in lumbar spine of patients symptomatic for low back pain. Eur Spine J.

[4] Li Z, Yang H, Liu M (2018). Clinical characteristics and risk factors of recurrent lumbar disk herniation: a retrospective analysis of three hundred twenty-one cases. Spine J.

[5] Wang H, Zhou Y (2016). Facet tropism: possible role in the pathology of lumbar disc herniation in adolescents. J Neurosurg Pediatr.

[6] Huang X, Ye L, Liu X (2020). The Relationship Between Facet Tropism and Cervical Disc Herniation. J Anat.

[7] Rong X, Wang B, Ding C (2017). The biomechanical impact of facet tropism on the intervertebral disc and facet joints in the cervical spine. Spine J.

[8] Xu C, Lin B, Ding Z, Xu Y (2016). Cervical degenerative spondylolisthesis: analysis of facet orientation and the severity of cervical spondylolisthesis. Spine J.

[9] Adams MA, Hutton WC (1980). The effect of posture on the role of the apophysial joints in resisting intervertebral compressive forces. J Bone Joint Surg.

[10] Noren R, Trafimow J, Andersson GB (1991). The role of facet joint tropism and facet joint angle in disc degeneration. Spine J.

[11] Huang X, Ye L, Wu Z: Biomechanical effects of lateral bending position on performing cervical spinal manipulation for cervical disc herniation: a three-dimensional finite element analysis. Evidence-Based Complementary and Alternative Medicine: ed 2 2018 pp416,418. 10.1155/2018/2798396 10.1155/2018/2798396 PMC6016226 29991954

[12] Ishihara H, Matsui H, Osada R, Ohshima H, Tsuji H (1997). Facet joint asymmetry as a radiologic feature of lumbar intervertebral disc herniation in children and adolescents. Spine.

[13] Karacan I, Aydin T, Sahin Z (2004). Facet angles in lumbar disc herniation: their relation to anthropometric features. Spine J.

[14] Pal GP, Routal RV, Saggu SK (2001). The orientation of the articular facets of the zygapophyseal joints at the cervical and upper thoracic region. J Anat.

[15] Kim HJ, Chun HJ, Lee HM (2013). The biomechanical influence of the facet joint orientation and the facet tropism in the lumbar spine. Spine J.

[16] Rong X, Liu Z, Wang B, Chen H, Liu H (2017). The facet orientation of the subaxial cervical spine and the implications for cervical movements and clinical conditions. Spine J.

[17] Cui JH, Kim YC, Lee K, Park GT, Kim KT, Kim SM (2018). Relationship Between Facet Joint Tropism and Degeneration of Facet Joints and Intervertebral Discs Based on a Histological Study. J Orthop.

[18] Eroglu A, Sarı E, Cüce F, Tok F, Atabey C, Düz B (2017). The Investigation of the Role of the Facet Joint Angle in the Development of L5–S1 Spondylolysis in Young Men. Turk J Phys Med Rehab..

[19] Lv B, Yuan J, Ding H (2019). Relationship Between Endplate Defects, Modic Change, Disc Degeneration, and Facet Joint Degeneration in Patients With Low Back Pain. Biomed Res Int..

[20] Song Q, Liu X, Chen DJ (2019). Evaluation of MRI and CT parameters to analyze the correlation between disc and facet joint degeneration in the lumbar three-joint complex. Medicine (Baltimore)..

[21] Okada E, Matsumoto M, Ichihara D (2009). Aging of the cervical spine in healthy volunteers: a 10-year longitudinal magnetic resonance imaging study. Spine J.

[22] Lee DY, Ahn Y, Lee SH (2006). The influence of facet tropism on herniation of the lumbar disc in adolescents and adults. J Bone Joint Surg Br..

